# Underutilized Medlar (*Mespilus germanica* L.) Fruit: Polyphenol Extraction Optimization, Chemical Profiling, and In Vitro Pharmacological Evaluation

**DOI:** 10.3390/plants15081169

**Published:** 2026-04-10

**Authors:** Nenad Mićanović, Nada Ćujić Nikolić, Jelena Živković, Katarina Šavikin, Nemanja Krgović, Jelena Popović-Đorđević

**Affiliations:** 1 University of Belgrade, Faculty of Agriculture, Nemanjina 6, 11080 Belgrade, Serbia; 2 Institute for Medicinal Plants Research “Dr. Josif Pančić”, Tadeuša Košćuška 1, 11000 Belgrade, Serbia; ncujic@mocbilja.rs (N.Ć.N.); jzivkovic@mocbilja.rs (J.Ž.); ksavikin@mocbilja.rs (K.Š.); nkrgovic@mocbilja.rs (N.K.)

**Keywords:** medlar fruit, extracts, polyphenols, RSM modeling, antioxidant activity, antidiabetic activity, isoquercitrin, rutin

## Abstract

Medlar (*Mespilus germanica* L.) fruit presents a good source of bioactive compounds. This study aimed to optimize the traditional extraction method, maceration, in order to obtain extracts rich in polyphenols. The total phenolic compounds (TPC) from physiologically ripe (PRMFs) and consumable ripe (CRMFs) medlar fruits were extracted to develop models with high accuracy and prediction capacity by response surface methodology (RSM). Furthermore, the main phenolic compounds in the extracts were quantified using HPLC, and the extracts were tested for antioxidant activity and hypoglycemic activity. The extracts were prepared according to a central composite design. The extraction parameters for both PRMFs and CRMFs were time (30–210 min), ethanol concentration (20–80%) and solid-to-solvent ratio (1:10–1:50). The obtained results indicated that the optimal conditions for the extraction were 210 min, 66.55% ethanol, and 1:50 solid-to-solvent ratio (PRMF), and 120 min, 74.96% ethanol, and 1:50 solid-to-solvent ratio (CRMF). Under the optimized conditions, values for TPC were in agreement with the values predicted by RSM. Isoquercitrin, rutin, procyanidin B2, chlorogenic acid and caffeic acid were the most abundant compounds in both PRMF and CRMF optimized extracts. TPC, antioxidant activity, and inhibition of α-glucosidase and α-amylase enzymes did not show significant differences (*p* > 0.05) among PRMF and CRMF extracts.

## 1. Introduction

Medlar (*Mespilus germanica* L.) is a long-living plant species that can grow in the form of a shrub or small tree (3–6 m). It belongs to the family Rosaceae [1]. The origin of the medlar plant is the area of the Caspian Sea, the Caucasus, and Northern Iran [2]. Nowadays, the species is mainly distributed in Turkey, Iran, and certain parts of Europe. The medlar fruit is round or pear-shaped, is yellow-green to dark brown in color, and has five large seeds and a cup at the top [3]. Harvesting medlar fruits at the physiological ripening stage, and storing them until the consumable ripe stage is a traditional practice that continues to this day [4]. During that period, the fruits change color to light brown, while the flesh softens. Chemically, the maturation process brings an increase in sugars and a decrease in tannins. Flesh browning is associated with the enzyme polyphenol oxidase, and softening with the breakdown of pectin. During the ripening process, the content of biologically active compounds such as vitamin C and polyphenols typically decreases. After the ripening process, medlar fruits have a limited shelf life and significant changes in texture, color, and flavor [1,4,5,6,7]. From a nutritional point of view, medlar fruit is a good source of macronutrients (carbohydrates and organic acids) and micronutrients (vitamins and essential elements) [8,9].

Medlar fruit is a good source of polyphenols such as flavonoids (quercetin, rutin, catechin, epicatechin, kaempferol, kaempferol-3-*O*-glucoside, quercetin-3-*O*-rhamnoside, pinocembrin), stilbenes (resveratrol), phenolic aldehydes (vanillin), and several phenolic acids (*p*-coumaric acid, protocatechuic acid, cholorgenic acid, gallic acid, caffeic acid) [9,10,11]. During the process of fruit ripening, there are complex changes in the composition of bioactive compounds, whereby the concentrations of individual phenolic compounds can increase, decrease or remain stable [12,13]. Unripe fruits generally have higher levels of certain polyphenols, especially flavonoids and tannins, which contribute to astringency and bitterness. Consumable ripe fruits show a decrease in tannins and some flavonoids, while other phenolic compounds may increase or remain stable, leading to a sweeter taste, softer texture, and higher antioxidant availability [6]. In the study by Rop et al. on fruit maturation, quercetin and its glycosylated derivatives, such as gluco- and rhamnosides, were the most abundant flavonols, while immature fruits contain higher levels of flavones, which were not detected in ripe fruits [6]. Comparison of physiologically ripe and consumable ripe medlar fruits is important because biochemical changes occur during ripening, including a reduction in tannins and acids, and cell wall degradation may affect the extraction of bioactive compounds (Rop et al., 2011) [6]. From a practical perspective, although physiologically ripe fruits exhibit a longer shelf life due to their firmer structure, the higher content of astringent compounds can limit the applications of the extract because it can negatively affect sensory quality. On the other hand, consumable ripe fruits have a shorter shelf life, but the content of astringency compounds decreases during the ripening process, while the content of sugar and other aromatic compounds increases, making them tastier and more suitable for use in food and dietary supplements.

Polyphenols are natural secondary plant metabolites that are present in all parts of plants, especially in fruits and flowers. They contribute to the nutritive and organoleptic properties of fruits and vegetables, such as color, smell, and taste (most often they give a bitter and pungent taste to plants due to the interaction of polyphenols and glycoproteins from saliva) [14,15]. The composition of polyphenols in plants depends on the variety and different factors (degree of maturity, exposure to sunlight, temperature, mineral supplementation, etc.). Phenolic profile is an important parameter for characterizing different parts of fruits and vegetables [14]. Moreover, many research studies have shown that polyphenols have a positive impact in the prevention and treatment of chronic diseases due to their antioxidant [16,17], anti-inflammatory [18], and antidiabetic activity [10].

Previous studies on the phytochemical composition of medlar fruit have demonstrated its potential as an important source of biologically active compounds such as polyphenols. However, no studies have systematically investigated the optimal extraction conditions for these compounds at different ripening stages using statistical approaches such as response surface methodology (RSM). Previous studies have confirmed the effectiveness of applying RSM in the process of optimizing the extraction of polyphenolic compounds from different grape fruits [19]. Extraction factors such as temperature, time, solid/solvent ratio and solvent concentration can significantly influence the yield of polyphenols from fruits. We expect that optimizing these extraction conditions using RSM will maximize the yield of polyphenols from medlar fruits, and that the optimal conditions will vary depending on the stage of ripening. This study provides practical guidelines for the efficient extraction of bioactive compounds from medlar fruits at different ripening stages.

In most studies, the extraction of polyphenols from medlar fruit can be performed in a conventional way, where the most common extraction agents (solvents) are water, ethanol, methanol, hydrochloric acid, and acetone [1,6,10,20]. Plant or fruit extracts rich in polyphenols have diverse applications in the food, pharmaceutical, and cosmetic industries due to their antioxidant, anti-inflammatory, and anticarcinogenic properties [21,22].

Extraction of phenolic compounds from plants has been studied by many researchers. The most studied method of polyphenol extraction is the conventional method based on solid–liquid extraction with various solvents. The conventional extraction method can include extraction pretreatment operations by microwave, ultrasound, ultrahigh pressure, enzyme assistance, etc. [23]. The principles of the mentioned methods and the parameters affecting them, as well as a relative comparison between them, have been recorded in several reviews [24,25]. The type of solvent plays a major role in the quantitative extraction, and 60–80% ethanol in water seems to be the most promising solvent for most of the phenolic groups [26]. The extraction time and temperature have a major effect on the yield, especially in microwave- or ultrasound-assisted extraction [27]. Conventional extraction methods have their own disadvantages, particularly in terms of the long extraction time and the relatively large quantities of organic solvents used [28]. However, they are still used more, in terms of optimizing their conditions and in terms of industrial scale application [29]. The increasing demand of the food and pharmaceutical industry for reducing production costs requires advanced technologies that will reduce production costs and ensure the quality of the produced extracts. A combination of different sample pretreatment processing methods with other extraction techniques has proven to be a good opportunity for developing optimized conditions for the extraction of bioactive compounds such as polyphenols [30,31].

It should be emphasized that, currently, the only approved natural antioxidant for food in the European Union, according to Regulation (EC) No 1333/2008 [32], is a specific group of extracts from rosemary (*Rosmarinus officinalis*). The regulation lists specify ethanol, acetone, hexane, or supercritical CO_2_ as permitted solvents for the extraction of antioxidant compounds. It should also be emphasized that the choice of solvent for polyphenol extraction depends on the nature of the solid matrix (i.e., the raw material), the polarity of the target compounds, and the desired purity of the extracts [27].

The presented study aimed to optimize the traditional maceration extraction of total phenolic compounds (TPC) from physiologically ripe (PRMF) and consumable ripe (CRMF) medlar fruits, and to develop highly accurate predictive models using central composite design and response surface methodology (RSM). In addition, the major phenolic compounds in the obtained optimized extracts were identified and quantified using HPLC, while the antioxidant and hypoglycemic potential of the extracts was also evaluated.

## 2. Results and Discussion

### 2.1. RSM Extraction Modeling

**Figure 1:**
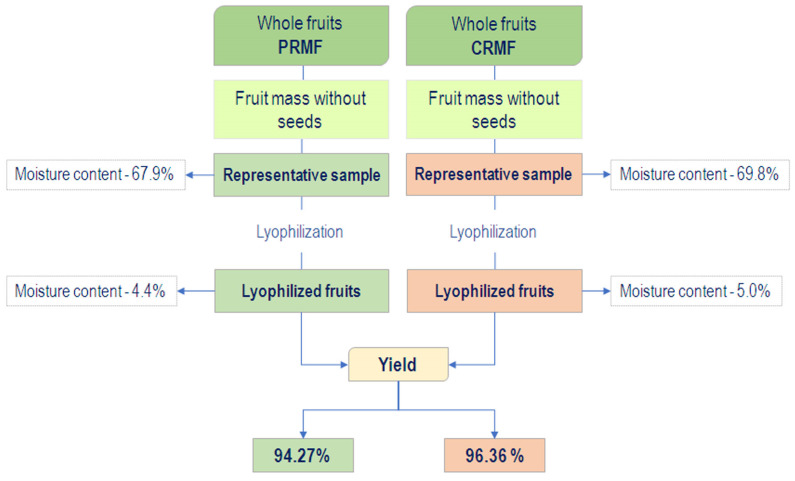
Drying efficiency of *M. germanica* fruits in two ripening stages: PRMF—physiologically ripe medlar fruit—and CRPM—consumable ripe medlar fruit.

Figure 1 illustrates the stages of sample preparation for polyphenol extraction, as well as drying efficiency, which reached 94.27% and 96.36% for the PRMF and CRMF, respectively.

The influence of three factors (independent variables), including time, ethanol concentration, and solid/solvent ratio, on the medlar fruit extraction was investigated to determine TPC. Results are presented in Table 1.

#### 2.1.1. Model Fitting

The analysis of variance (ANOVA) was performed to evaluate the quality of the fitted models and to identify significant factors and their interactions for the extraction of TPC from PRMF and CRMF (Table 2). The obtained second-order polynomial models for the extraction TPC were statistically significant (*p* < 0.05). The correlation coefficient (R^2^) was relatively high for TPC extracted from PRMF (0.9313) and CRMF (0.822), which indicates a good fit between experimental and predicted values. A non-significant lack of fit (*p* > 0.05) implies that the model adequately represents the actual relationship between the investigated factors and responses.

#### 2.1.2. Influence of Selected Factors on TPC

Based on the results obtained from the central composite design (CCD) the total phenolic compounds (TPC) in PRMF extracts varied between 0.99 and 2.14 mg/g of dry weight (dw), and the highest TPC was achieved by using 50% (*v*/*v*) ethanol for 210 min., while the solid-to-solvent ratio was 1:50. The lowest TPC value was obtained by combining 20% (*v*/*v*) ethanol for 210 min, while the solid-to-solvent ratio was 1:30 (Table 1). On the other hand, the content of phenolic compounds (TPC) extracted from CRMF varied between 0.69 and 2.18 mg/g dw, and the highest TPC was achieved by using 50% (*v*/*v*) ethanol for 30 min., while the solid-to-solvent ratio was 1:50. The lowest TPC value was obtained by combining 20% (*v*/*v*) ethanol for 120 min, while the solid-to-solvent ratio was 1:10 (Table 1).

For the extraction of TP from PRMF, all of the three investigated factors (A, B, and C) showed significant influence. Extraction time significantly affects polyphenol yield, meaning that an increase in extraction time leads to a higher TPC. This may be related to the fact that prolonged exposure of the sample to the solvent allowed compounds to migrate into the solvent from the plant material [33]. In the case of CRMF, the influence of factor time on polyphenol yield did not have a significant effect.

The polynomial equations for TPC were as follows:

PRMF = TPC (mg GAE/g dw) = 1.56 + 0.1241A + 0.297B + 0.3101C − 0.2812C^2^

CRMF = TPC (mg GAE/g dw) = 1.68 + 0.3405B + 0.2798C − 0.2046B^2^

**Figure 2:**
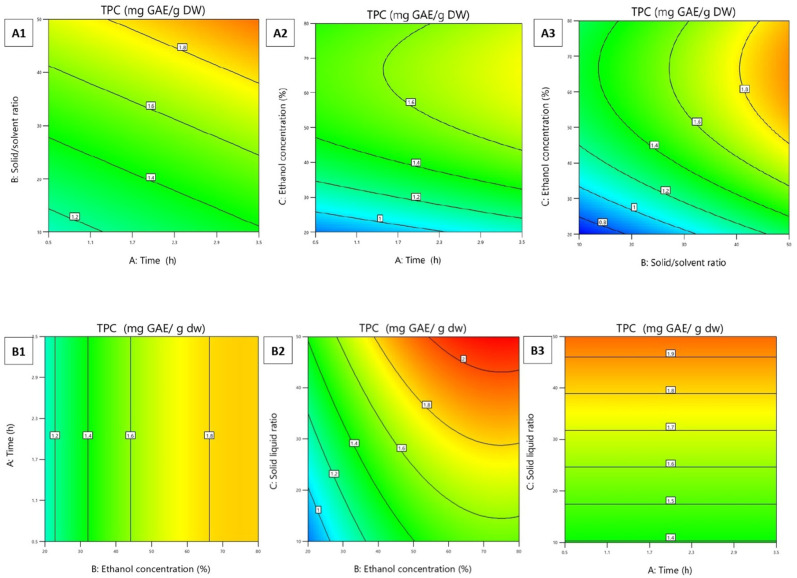
Contour plots of total phenolic content (TPC) for physiologically ripe medlar fruit, PRMF (**A1**–**A3**), and consumable ripe medlar fruit, CRMF (**B1**–**B3**).

This can be explained by the fact that prolonged extraction led to a decrease in phenolic content of extracts, as oxidation of the compounds could occur with the increasing time of extraction [34]. The study of the effect of different solvent concentrations on the extraction efficiency showed that the maximum TPC was achieved for both studied *M. germanica* fruits with an ethanol concentration of 50%. Water is important for the swelling of plant material, while ethanol is responsible for breaking the bond between solutes and the plant matrix, thus enabling better mass transfer of the compound [35]. On the other hand, a high percentage of ethanol can cause protein denaturation, causing an increase in the viscosity of the plant matrix, and thus preventing the release of phenols [30]. The effect of solvent volume on the extraction yield of TPC showed that the highest TPC values are achieved by applying the solid-to-solvent ratio of 1:50 for both medlar fruits. As the amount of solvent increases, the possibility of contact between the bioactive components and the extraction solvent (causing better extraction of compounds from the extracted cell wall) increases. Higher solvent volumes reduce viscosity and improve the movement (mass transfer) of phenolic compounds into the liquid phase, increasing extraction efficiency. However, the increase in the ratio of solids to solvents will not continue to increase phytochemical content extracted when equilibrium is reached [36]. During ripening, the fruit softens, which is caused by changes in cell turgor and the primary structure of the cell wall. Most of the polysaccharide components of the cell wall undergo some degree of controlled degradation, resulting in loosening and swelling of the wall structure and weakening the strength of the cell wall [37]. This may explain why a higher TPC was achieved in less time in CRMF. The cell wall that is weakened during the ripening process breaks down in less time than in fruits where this process has not begun. To define optimal conditions for TPC, a contour plot was designed for PRMF (Figure 2A1–A3) and CRMF (Figure 2B1–B3) extracts.

#### 2.1.3. RSM Optimization of the Extraction Process and Model Validation

The optimal extraction conditions based on statistical analysis were, for PRMF: time: 210 min; ethanol concentration: 66.55%; and solid-to-solvent ratio: 1:50. Meanwhile, for CRMF, they were: time: 120 min; ethanol concentration: 74.96%; and solid-to-solvent ratio: 1:50. Under these conditions, the experimentally obtained results for TPC were 1.95 mg GAE/g dw for PRMF and 2.52 mg GAE/g dw for CRMF, respectively. It was found that the mean values of the experimentally obtained results were in close agreement with the predicted values, and these results confirmed the suitability of the developed quadratic model (Table 3). The good correlation between the predicted and experimental values indicates that the response surface methodology was successfully applied for the extraction of polyphenolic compounds from medlar fruits. To determine the adequacy of the predictive model, experiments were conducted in five replicates under previously defined optimal conditions, and the results are shown in Table 3. The verification process was completed under conditions that were estimated as optimal by RSM. Similar to results obtained in the present study, Ilaiyaraja et al. reported that 62.7% ethanol concentration was optimal in the extraction of total phenolics from *Feronia limonia* (wood-apple) fruit [38]. For the extraction of polyphenols from mulberry fruit, Kostić et al. found an optimal ethanol concentration of 60–80% and an optimal time of 233 min [39]. A study conducted by Brahmi et al. showed that the highest TPC yield was at a liquid-to-solid ratio of 1:50 from potato peels [40].

From a practical perspective, the optimized extraction conditions identified in this study could be directly applied in the development of functional ingredients or dietary supplements, particularly those aimed at delivering natural bioactive compounds such as polyphenols.

**Figure 3:**
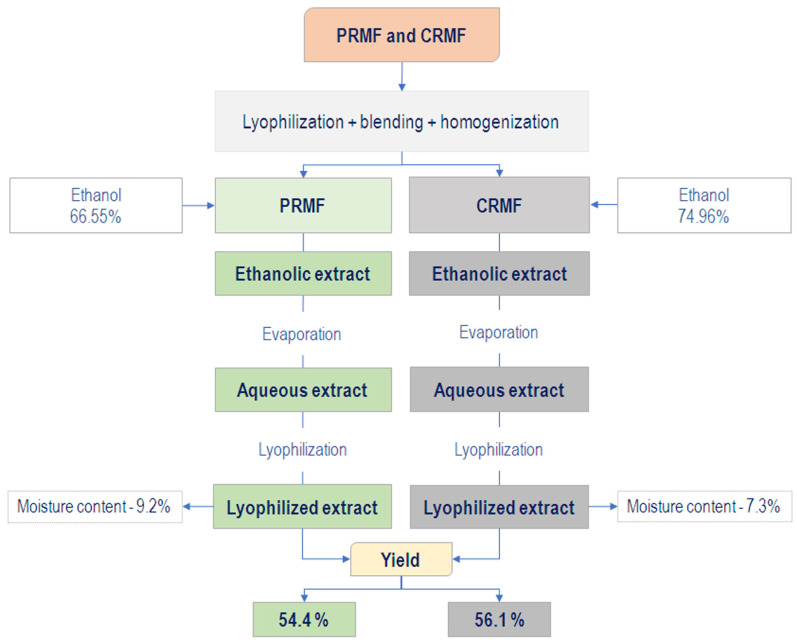
Extraction yield of *M. germanica* fruits in two ripening stages: PRMF—physiologically ripe medlar fruit—and CRMF—consumable ripe medlar fruit.

Figure 3 illustrates the stages of obtaining the optimal lyophilized extracts, as well as their yield, which were 54.4% and 56.1% for PRMF and CRMF, respectively. A higher extract yield indicates a higher extraction efficiency, which reflects a higher amount of soluble compounds transferred from the plant material to the solvent under the applied experimental conditions. Extraction efficiency is expressed as a percentage of the total extract obtained relative to the initial mass of plant material.

The demonstrated efficiency of maceration under optimized conditions confirms that traditional extraction techniques, when properly adjusted, can provide high yields of polyphenols from *Mespilus germanica*, offering a cost-effective and accessible approach.

### 2.2. Total Phenolic Content and HPLC Analysis

Identification and quantification of the most dominant phenolic compounds in the optimized lyophilized extracts obtained using maceration of both pre-ripe medlar (PRMF) and consumable ripe medlar (CRMF) fruits were done using HPLC, and the results are summarized in Table 4. The total phenol content (TPC) in the optimized lyophilized extracts was significantly higher in the PRMF extract compared to the CRMF extract (*p* < 0.05, Table 4). Previous reports have identified cinnamic acid derivatives (such as chlorogenic, caffeic, *p*-coumaric, and ferulic acids) and flavonoid compounds (including rutin, quercetin, and epicatechin gallate) as the major phenolic constituents of medlar fruits [10,20,41].

In the present study, chlorogenic acid and caffeic acid were the predominant phenolic acids. Contents of hyperoside, quercetin, and caffeic acid were not significantly different between the samples (*p* > 0.05). These findings are consistent with the observations of Gruz et al. [41], who reported a decline in free phenolic acids during fruit ripening, accompanied by an increase in ester-linked phenolic forms at intermediate stages. Such changes reflect the complex biochemical transformations occurring during ripening, which influence both the levels and composition of bioactive compounds. Results of the flavonoid fraction confirmed the presence of quercetin, rutin, hyperoside, epicatechin, procyanidin B, and isoquercitrin in both PRMF and CRMF ethanol extracts. Overall, quercetin and hyperoside were detected in lower concentrations compared to rutin and isoquercitrin. The concentrations of rutin, chlorogenic acid and epicatechin were significantly higher in the CRMF extract, whereas isoquercitrin and procyanidin B2 were more abundant in the PRMF extract (*p* < 0.05). Nikolić et al. also found higher epicatechin concentrations in later stages of medlar fruit ripening (1.9 mg/g fresh weight 121 days after flowering), and the procyanidin B concentration increased up to 121 days after flowering (2.1 mg/g fresh weight), while decreasing (1.8 mg/g fresh weight) in the later stage 162 days after flowering [20]. Among all quantified phenolic compounds, isoquercitrin was the most dominant, with concentrations of 140.96 μg/g and 114.67 μg/g in PRMF and CRMF extracts, respectively. These results align with the findings of Nikolić et al., who reported higher isoquercitrin levels compared to rutin [20]. In the same study, the authors observed that isoquercitrin content increased from day 23 to day 96 of fruit development, after which it began to decline. In contrast, Katanić-Stanković et al. [10] reported chlorogenic acid as the predominant compound in *M. germanica* ethanolic extracts, with concentrations reaching 78.8 mg/kg. Similarly, Tessa et al. [9] identified rutin as the main flavonoid in medlar fruit extracts, with a content of 12.5 mg/100 g. In comparison with these literature data, the concentrations obtained in the present study were lower, which may be attributed to differences in fruit maturity, geographical origin, or extraction conditions. The presence of key phenolic compounds, such as isoquercitrin, rutin, and chlorogenic acid, further supports the potential application of medlar extracts in developing new functional products.

### 2.3. Pharmacological Activities

#### 2.3.1. Antioxidant Activity

Bioactive compounds such as polyphenols have positive effects on body health by mitigating the effects of oxidative stress through antioxidant activity. The results obtained for antioxidant activity of optimized and lyophilized PRMF and CRMF extracts, evaluated by DPPH, ABTS^+^, and FRAP assays, are presented in Table 5.

Extracts of both physiologically ripe fruits (PRMFs) and consumable ripe medlar fruits (CRMFs) exhibited comparable antioxidant activity, with no statistically significant difference (*p* > 0.05). For comparison, in the study conducted by Nikolić et al., a higher scavenging capacity of DPPH^·^ free radicals was reported for the extract obtained from the medlar fruit in the early ripening stadium (121 days) compared to the extract obtained from the fruit in the advanced ripening stadium (162 days) [20]. The FRAP assay, which is based on the measurement of the ability of the substance to reduce Fe^3+^ to Fe^2+^, has been frequently used for a rapid evaluation of the total antioxidant capacity of various foods and beverages, and also different plant extracts [41]. The comparison of the antioxidant activity of the tested extracts determined by the FRAP method showed similar reducing capability of the extracts. Vitamin C, which was used as the standard, demonstrated the highest antioxidative activity in all the performed in vitro assays. Studies conducted by Tzulker et al. [42], as well as Gruz et al. [41], have shown that antioxidant activity is related to polyphenol content in the extract. It was reported that polyphenol content was higher (170 mg gallic acid equivalents/100 g fresh matter—GAE/100 g FM) in the initial stage of fruit ripening (134 days after full bloom), while during the ripening period (174 days after full bloom) it decreased (93 mg GAE/100 g FM) [41]. The results reported by the same authors showed that the phenolic content is significantly correlated with the ABTS^+^ test [41]. The stage of fruit ripeness does not always significantly affect the antioxidant activity of extracts, as changes in individual phenolics during fruit ripening compensate for each other, and the dominant bioactive compounds remain relatively stable. Although the concentrations of individual phenolic compounds and flavonoids may change during ripening, the overall antioxidant activity often remains relatively stable due to compensatory effects among the components [43,44].

#### 2.3.2.  Hypoglycemic Activity

**Figure 4:**
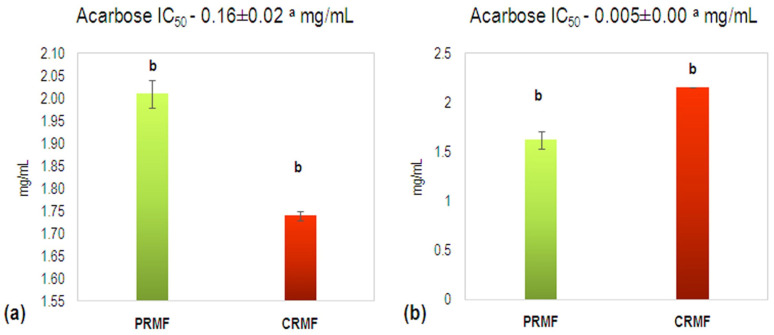
Inhibitory activity toward (**a**) α-glucosidase and (**b**) α-amylase of physiologically ripe medlar fruit (PRMF) and consumable ripe medlar fruit (CRMF) optimized lyophilized extracts, and the standard drug acarbose. Results are presented as mean ± standard deviation. Mean values (*n* = 3) followed by different letters differ significantly at *p* < 0.05.

Diabetes mellitus has become a major public health threat across the globe. α-Amylase and α-glucosidase are the main enzymes involved in the breakdown of sugars in the human body [45]. Slowing down glucose release and absorption plays a key role in the management of diabetes mellitus type 2. Studying enzyme inhibition is biologically important because many natural regulators and drugs work by inhibiting enzymes. Strong inhibition may indicate that the tested compound could potentially regulate biochemical pathways or serve as a candidate for therapeutic applications [46]. Inhibition of α-amylase and α-glucosidase enzymes is an effective therapeutic approach, and inhibitors of these enzymes are potential targets in the development of medications for diabetes treatment. Polyphenols can inhibit the enzymes α-amylase and α-glucosidase through different mechanisms. In α-amylase, inhibition occurs competitively, where polyphenols bind to the active site of the enzyme, as well as through protein interactions that change the conformation of the enzyme or bind the substrate (starch). In α-glucosidase, inhibition can be competitive, non-competitive, or a combination, where polyphenols prevent the binding of disaccharides and, by changing the structure of the enzyme, reduce its activity [47]. In this study, the hypoglycemic potential of the optimized and lyophilized PRMF and CRMF extracts was investigated through an in vitro analysis of α-amylase- and α-glucosidase-inhibitory activities. The obtained results (Figure 4) showed that CRMF extract had stronger α-glucosidase-inhibitory activity (IC_50_—1.74 mg/mL), while in the case of α-amylase inhibition, stronger activity was exhibited by PRMF extract (IC_50_—1.62 mg/mL), but with no significant difference (*p* > 0.05). This may be explained by compensatory changes among individual phenolic compounds, which keep the total bioactive content relatively stable, maintaining a similar overall biological activity. Both medlar extracts showed significantly lower α-glucosidase- and α-amylase-inhibitory activity (IC_50_: 0.16 mg/mL and 0.005 mg/mL, respectively) than the commercial drug acarbose (*p* < 0.05).

However, in the study conducted by Katanić Stanković et al. [10], the ethanolic extract of medlar fruit showed comparable α-glucosidase-inhibitory activity (IC_50_—199.84 µg/mL) to the standard drug acarbose (IC_50_—201.38 µg/mL). It is reported that the medlar bud and fruit extracts showed inhibition activity against α-amylase (35% of α-amylase inhibition of medlar fruit extract at 100 µg/mL concentration) [48]. The authors reported that the inhibitory effect could be attributed to the phenolic acids of the medlar plant. Phenolic acids such as caffeic acid, ferulic acid, syringic acid, ellagic acid, chlorogenic acid, and other phenolic compounds such as quercetin and vanillin may be responsible for the inhibition of α-amylase and α-glucosidase [48]. The obtained results of this study can be attributed to the presence and content of specific phenolic compounds such as isoquercitrin, rutin, caffeic acid, procyanidin B2, and chlorogenic acid in PRMF and CRMF extracts.

The lack of significant differences in total phenolic content, antioxidant activity, and enzyme inhibition between physiologically and consumable ripe fruits means that the fruits can be used at different stages of ripeness, making their industrial use easier by reducing limitations related to fruit maturity.

## 3. Materials and Methods

### 3.1. Plant Material

**Figure 5:**
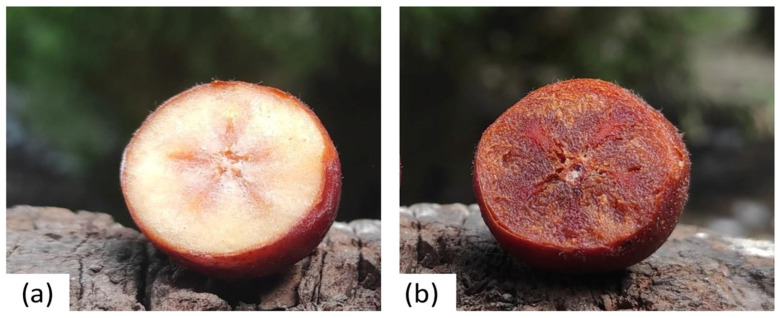
Cross-section of medlar (*M. gemanica* L.) fruits: (**a**) Physiologically ripe medlar fruit (PRMF) and (**b**) consumable ripe medlar fruit (CRMF) (Photos by N. Mićanović).

Medlar fruits of the ‘Domestic medlar’ variety [49] were obtained from a local producer (plantation in Central Serbia near Mali Požarevac; 49°34′52.6″ N; 47°13′49″ E). Fruits were harvested as physiologically ripe fruits (PRMF) (Figure 5a) and then left in a home cellar to soften until ripeness in the consumable stage, in an uncontrolled atmosphere, at a temperature of 10 °C for 21 days (consumable ripe fruits—CRMFs) (Figure 5b). Samples of PRMFs and CRMFs were washed, and seeds were removed from the flesh. The skin with the pulp was blended and homogenized, and then it was placed in a deep freeze for the lyophilization process. Medlar samples were lyophilized in a Beta 2–8 LD plus model lyophilizer (Christ, Osterode am Harz, Germany). After the lyophilization process, the samples (with water content ≤ 5%) were ground in the electric mill into “breadcrumbs” form (≤4 mm). Lyophilized and powdered samples were packed into glass jars with parafilm foil and left in a cold and dry place until further analysis.

### 3.2. Reagents and Standards

All chemicals used as reagents or standards were of analytical grade: ethanol 96% (*v*/*v*) (Honeywell Riedel de Haën, Seelze, Germany), Folin–Ciocalteu phenol reagent (Sigma-Aldrich, Steinheim, Germany), sodium carbonate, methanol, and orthophosphoric acid were obtained from Sigma–Aldrich Chemie GmbH, Münich, Germany; acetonitrile (Merck, Darmstadt, Germany) was HPLC grade; and ultra-pure water was prepared using a Milli-Q purification system (Millipore, Guyancourt, France). The following standards were used: gallic acid (Extrasynthese, Cedex, Genay, France), rutin, isoquercitrine, epicatechin, hyperoside, chlorogenic acid, and procyanidin B2 (all from ChemFaces, Wuhan, China).

### 3.3. Drying Efficiency

Drying process yield (Y) was calculated as the ratio between dried samples (PRMF/CRMF) mass (g) and the expected mass after the drying process:

(1) Y (%) = m dried samples/m expected dried samples × 100

Expected mass calculation was based on the sum of the dry residue in PRMF/CRMF before the drying process, multiplied by the mass of PRMF/CRMF used for the drying process:

(2) m expected (g) = m dry residue × m PRMF/CRMF

Initially, a mass of 1100 g of whole *M. germanica* fruits was taken, for both stages of ripening. Then, the pits were removed from the whole fruits, and masses of pitted fruits (pulp with skin) were obtained for PRMF, 861 g, and for CRMF, 780 g. From that total cleaned mass (pulp with skin), for representative samples, masses of 292.28 g for PRMF and 211.90 g for CRMF were taken.

### 3.4. Moisture Content

The moisture content of the obtained samples was determined gravimetrically using a Moisture Analyzer (Kern & SOHN Moisture Analyzer GmbH, Balingen, Germany). All samples were dried at 105 °C until a constant weight, and the moisture content was calculated from the difference in mass before and after drying. All measurements were made in triplicate, and the results are expressed as percentages (%, *w*/*w*).

### 3.5. Extraction Procedure and Response Surface Methodology

The extraction of the *M. germanica* fruits was performed using the traditional maceration extraction method, in a 50 mL Erlenmeyer flask, mixing on a laboratory shaker (Unimax 1010, Heidolph, Schwabach, Germany). The rotation of the shaker was fixed at 200 rpm throughout the experiment, and all extractions were performed at room temperature. The samples of PRMFs and CRMFs (0.5–2.5 g) were placed in the Erlenmeyer flask, with a certain amount of solvent (25 mL), to achieve a given solid-to-solvent ratio. For the preparation of *M. germanica* fruit extracts according to the central composite design, extraction was performed with different A: time (30, 120, and 210 min); B: concentration of ethanol (20, 50, and 80% *v*/*v*); and C: solid/solvent ratio (1:10, 1:30, and 1:50).

The extraction procedure of medlar fruits is based on the central composite design (CCD), with three independent variables (extraction time, ethanol concentration, and solid-to-solvent ratio), which were varied at three levels. The design consisted of 21 randomized runs with three replicates at the central point. The quadratic (polynomial) model proposed for the response was as follows:

$$Y_{k}=b_{k0}+\sum_{i=1}^{4}b_{ki}X_{i}+\sum_{i=1}^{4}b_{kii}X_{i}+\sum_{i=1}^{3}\sum_{j=i+1}^{4}b_{kij}X_{i}X_{j}$$

where Y_k_ is the predicted response, b_k0_ is the constant coefficient, b_ki_ is the linear coefficient, b_kii_ is the quadratic coefficient, and b_kij_ is the interaction coefficient of variables, and X is the coded independent variable. For the evaluation of the significance of independent variables and the significance of their interactions (*p* < 0.05), analysis of variance (ANOVA) was used. To obtain the coefficients and final equations, which can be used to approximate the response, a multiple regression analysis was used. The best-fitting quadratic models were generated through multiple linear regressions with backward elimination. Therefore, non-significant factors (*p* > 0.05) were excluded from the model, but those factors that were necessary to maintain the hierarchy of the model are retained. The adequacy of the model was determined by evaluating the lack of fit, coefficient of determination (R^2^), and *p*-value of the model. The desirability function approach was used for the optimization of the extraction. This numerical optimization technique represents the desirability degree of the response with respect to the target value, intended to be reached [50,51]. The software Design Expert (trial version, Stat-Ease, Minneapolis, MN, USA) was used for experimental design, data analysis, and determination of optimal conditions. Extraction under optimal conditions was performed in three replicates to validate the obtained model.

### 3.6. Lyophilization of Optimal Extracts

Initially, masses of 20.01 g of homogenized lyophilized PRMF and 20.37 g of homogenized lyophilized CRMF were taken. Then, ethanol was added as a solvent in an amount of 1000 mL in a concentration 66.5% for PRMF and 75% for CRMF. After maceration and filtration, an amount of 950 mL ethanolic extract was obtained for both PRMF and CRMF. After evaporation of ethanol, the amount of aqueous extract was 220 mL for PRMF and 250 mL for CRMF. Drying by using the lyophilization process (Lyophilizer Christ, Beta 2–8, LD Plus, Martin Christ, Osterode am Harz, Germany), a mass of 11.99 g of lyophilized PRMF extract and a mass of 12.32 g of lyophilized CRMF extract were obtained. The moisture content was 9.2% for the lyophilized PRMF extract and 7.3% for the lyophilized CRMF extract. The obtained optimized lyophilized extracts were used for HPLC analysis and pharmacological tests.

### 3.7. Total Phenolic Content

The total phenolic content (TPC) in the extracts was determined by a modified Folin–Ciocalteu method [52]. Briefly, 200 μL of extracts were added to 1 mL of 1:10 diluted Folin–Ciocalteu reagent. After 4 min, 800 μL of sodium carbonate (75 g/L) was added. After 2 h of incubation at room temperature in the dark, the absorbance at 765 nm was measured by a spectrophotometer (Agilent Cary, Agilent Technologies, Santa Clara, CA, USA). Gallic acid (0–100 mg/L) was used for calibration of a standard curve (A = 0.10281 × C, r > 0.99). The results were expressed as milligrams of gallic acid equivalents per gram of dry weight of medlar fruit (mg GAE/g dw) and dry weight of lyophilized extract (mg GAE/g dwle). All measurements were performed in three replicates, and results were presented as their mean value. The measurement was done in three repetitions.

### 3.8. HPLC Analysis

Quantification of dominant phenolic compounds was carried out on an Agilent 1260 RR high-performance liquid chromatography (HPLC) system (Agilent, 1260 Infinity II, Waldbronn, Germany) equipped with a diode array detector (DAD) scanning from 190 to 550 nm. Separation was achieved on a Zorbax SB-C18 reversed-phase column (150 mm × 4.6 mm i.d., 5 μm particle size; Agilent). The mobile phases consisted of (A) water containing 1% (*v*/*v*) orthophosphoric acid and (B) acetonitrile. The gradient program was as follows: 0–2.6 min, 90–85% A; 2.6–8 min, 85% A; 8–10.8 min, 85–80% A; 10.8–18 min, 80% A; 18–23 min, 80–70% A; 23–25 min, 70–50% A; 25–27 min, 50–30% A; 27–29 min, 30–10% A; 29–31 min, 10–0% A; and 31–34 min, 0% A. The detection wavelengths were set at 260, 280, 320, and 360 nm. The flow rate was maintained at 0.8 mL/min, with an injection volume of 8 μL, and the column temperature was held constant at 40 °C. Compound identification was based on comparison of retention times and UV spectra with those of authentic standards. Quantification was carried out using calibration curves, and the concentrations were expressed as micrograms per gram of dry weight lyophilized extract (μg/g dwle) [53]. The measurement was done in three repetitions. Relevant chromatograms are presented in the Supplementary Materials (Figures S1–S4).

### 3.9. Antioxidant Activity

#### 3.9.1. DPPH Assay

The ability of *M. germanica* fruit extracts to neutralize 2,2-diphenyl-1-picrylhydrazyl (DPPH) radicals was evaluated using the method of Krgović et al. [54]. Serial dilutions of extracts in 60% ethanol (stock solution concentrations of PRMF extract 89.73 mg/mL, and CRMF extract 90.03 mg/mL; 2 mL) were mixed with 0.5 mM DPPH solution in ethanol (0.5 mL), strongly shaken, and left in the dark for 30 min. The absorbance was measured at 517 nm, using 60% ethanol as a blank (Spectrophotometer Agilent Cary, Agilent Technologies, USA). The percentage of DPPH radical inhibition was calculated using the following formula:

Inhibition (%) = [(Ac − As)/Ac] × 100

where Ac is the absorbance of the control (60% ethanol was used instead of the extract solution), and as is the absorbance of the sample. The results are expressed as IC_50_ values (mg/mL). Ascorbic acid was used as a positive control. Testing was done in three repetitions.

#### 3.9.2. FRAP Assay

Assessment of *M. germanica* extracts’ total antioxidant capacity, based on their ability to reduce ferric (Fe^3+^) to ferrous (Fe^2+^) ion, was performed following the method described by Benzie and Strain [55], with slight modifications. Briefly, FRAP reagent was prepared ex tempore by mixing 1 mL of a TPTZ solution (10 mmol/L of 2,4,6-tripyridyl-s-triazine in 40 mmol/L of hydrochloric acid), 1 mL of the FeCl_3_ × 6H_2_O solution (20 mmol/L in distilled water), and 10 mL of acetate buffer (300 mmol/L, pH 3.6). The extracts dissolved in 60% ethanol in the appropriate concentration (stock solution concentrations of PRMF extract 13.92 mg/mL, and CRMF extract 13.77 mg/mL; 0.1 mL) were added to FRAP reagent (3 mL), and incubated for 30 min at 37 °C. The absorbances were measured at 593 nm against a blank (3 mL of FRAP reagent mixed with 0.1 mL of 60% ethanol) (Spectrophotometer Agilent Cary, Agilent Technologies, USA). For the construction of a calibration curve, different concentrations of FeSO_4_ × 7H_2_O water solutions (200–1000 μmol/L) were used, resulting in the calibration equation y = 0.0209x + 0.0015. The FRAP value was expressed as μmol Fe^2+^/g of dry extract. Ascorbic acid was used as a positive control. Testing was done in three repetitions.

#### 3.9.3. ABTS^+^ Assay

The ability of *M. germanica* fruit extracts to neutralize 2,2′-azino-bis(3-ethylbenzothiazoline-6-sulfonic acid) (ABTS^+^) free radicals was evaluated according to the adopted procedure of Re et al. [56]. The ABTS^+^ basic solution was prepared from 14 mM ABTS^+^ and 4.9 mM K_2_S_2_O_8_ solutions in water, which were mixed in a ratio of 1:1 (*v*/*v*), and kept in the dark at room temperature for 16 h. Before the assay, ABTS^+^ solution was diluted with water until an absorbance of 0.700 was reached at 734 nm (Spectrophotometer Agilent Cary, Agilent Technologies, USA). Properly diluted extracts in 60% ethanol (stock solution concentrations of PRMF extract 5.38 mg/mL, and CRMF extract 5.40 mg/mL; 100 μL) were reacted with diluted ABTS^+^ solution (900 μL) in test tubes for 7 min at 30 °C in the dark, and the absorbances were measured at 734 nm, against water as a blank. The percentage of ABTS^+^ radical inhibition was calculated using the formula:

Inhibition (%) = [(Ac − As)/Ac] × 100

where Ac is the absorbance of the control (60% ethanol was used instead of the extract solution), and As is the absorbance of the sample. The results are expressed as IC_50_ values (mg/mL). Ascorbic acid was used as a positive control. Testing was done in three repetitions.

### 3.10. Hypoglycemic Activity

#### 3.10.1. α-Glucosidase Inhibition Assay

α-Glucosidase-inhibitory activity of *M. germanica* fruit extracts was evaluated according to the method reported by Indrianingsih et al. [57], with slight modifications. Properly diluted extracts in 60% ethanol (stock solution: physiologically ripe medlar fruit extract 75.92 mg/mL, consumable ripe medlar fruit extract 76.47 mg/mL; 30 μL) were mixed with *p*-nitrophenyl-α-D-glucopyranoside solution (250 μL) and phosphate buffer pH 6.9 (470 μL); the formed mixtures were pre-incubated at 37 °C for 5 min. Then, α-glucosidase solution (250 μL) was added, and the incubation continued for 15 min. The reaction was stopped by adding a 0.20 M Na_2_CO_3_ solution (1000 μL). The absorbances were measured at 400 nm against a blank (the enzyme solution was replaced with phosphate buffer, pH 6.9) (Spectrophotometer Agilent Cary, Agilent Technologies, USA). The percentage inhibition of enzyme activity was calculated using the following formula:

Inhibition (%) = [(Ac − As)/Ac] × 100

where Ac is the absorbance of the control (the extract solution was replaced with 60% ethanol) and As is the absorbance of the extracts. The results are expressed as IC_50_ values (mg/mL), which were obtained using the linear regression analysis. Acarbose was used as a positive control. Testing was done in three repetitions.

#### 3.10.2. α-Amylase Inhibition Assay

α-Amylase-inhibitory activity of *M. germanica* extracts was evaluated according to the method reported by Ahmed et al. [58], with slight modifications. Properly diluted extracts in 60% ethanol (stock solution concentrations: physiologically ripe medlar fruit (PRMF) extract, 12.41 mg/mL; consumable ripe medlar fruit (CRMF) extract, 12.43 mg/mL; 200 μL) were mixed with α-amylase solution (200 μL) and incubated at 37 °C for 15 min. The same volume of 1.0% *w*/*v* starch solution (200 μL) was added, and incubation was continued for 10 min. Finally, DNS (3,5-dinitrosalicylic acid) solution (200 μL) was added, the mixtures were covered, and kept for 15 min in a boiling water bath. The absorbances were measured at 540 nm against a blank (the enzyme solution was replaced with phosphate buffer, pH 6.9) (Spectrophotometer Agilent Cary, Agilent Technologies, USA). The percentage inhibition of enzyme activity was calculated using the following formula:

Inhibition (%) = [(Ac − As)/Ac] × 100

where Ac is the absorbance of the control (the extract solution was replaced with 60% ethanol) and As is the absorbance of the extracts. The results are expressed as IC_50_ values (mg/mL). Acarbose was used as a positive control. Testing was done in three repetitions.

## 4. Conclusions

This study evaluated the potential of medlar (*M. germanica* L.) fruit extracts through the characterization of polyphenolic compounds and the assessment of their antioxidant and hypoglycemic activities. For the first time, the maceration technique was optimized for both physiologically ripe (PRMFs) and consumable ripe (CRMFs) fruits using response surface methodology. The significant factors for PRMFs were time, ethanol concentration, and solid/solvent ratio, while for CRMFs, the influence of time was not shown to be significant on the polyphenol yield. The optimization experiment showed that the best conditions for obtaining a high yield of total phenols were: 210 min, 66.55% ethanol concentration, and 1:50 solid-to-solvent ratio for PRMF; and 120 min, 74.96% ethanol concentration, and 1:50 solid-to-solvent ratio for CRMF. HPLC analysis showed that concentrations of isoquercitrin and procyanidin B2 were significantly higher in PRMF; rutin and epicatechin were higher in CRMF, whereas TPC was higher in PRMF (*p* < 0.05). The ripening stage had no significant effect on the polyphenol content or biological activities, indicating that medlar fruit can be utilized effectively at different maturation stages. The study showed that the applied factorial design is an adequate model for optimizing the extraction process. The adequacy of the model obtained by RSM was demonstrated by the close agreement between the predicted and observed values. The obtained results also indicated that maceration can be a simple and effective method for the extraction of phenolic compounds from medlar fruits. The study provides a scientific basis for the potential use of *M. germanica* fruit extracts in functional foods and nutraceuticals, highlighting their practical and industrial relevance.

On the other hand, the limitations are reflected in the fact that only one medlar variety is used in the experiment. Moreover, the study focused on time, ethanol concentration, and solids-to-solvent ratio as extraction parameters, while other factors that may influence the extraction process, such as temperature and particle size of the plant material, were not examined.

## Figures and Tables

**Table 1:** Central composite design for physiologically ripe medlar fruit and consumable ripe medlar fruit with extraction parameters, and experimentally observed response for total phenolic content.

		Factor 1	Factor 2	Factor 3	Response
Std	Run	A: Time (h)	B: Ethanol Concentration (%)	C: Solid/Solvent Ratio	TPC ^1^ (mg GAE/g dw) ^2^
Physiologically ripe medlar fruit (PRMF)					
1	9	0.5	20	30	1.00
2	13	3.5	20	30	0.99
3	10	0.5	80	30	1.52
4	15	3.5	80	30	1.73
5	11	0.5	50	10	1.13
6	6	3.5	50	10	1.35
7	17	0.5	50	50	1.56
8	5	3.5	50	50	2.14
9	3	2	20	10	0.68
10	8	2	80	10	1.23
11	14	2	20	50	1.20
12	4	2	80	50	1.86
13	12	2	50	30	1.64
14	16	2	50	30	1.53
15	2	2	50	30	1.49
16	1	2	50	30	1.70
17	7	2	50	30	1.48
18	9	0.5	20	30	1.56
19	10	0.5	80	30	2.05
20	11	0.5	50	10	1.48
21	17	0.5	50	50	1.97
Consumable ripe medlar fruit (CRMF)					
1	9	0.5	20	30	1.22
2	13	3.5	20	30	1.17
3	10	0.5	80	30	1.63
4	15	3.5	80	30	2.11
5	11	0.5	50	10	1.27
6	6	3.5	50	10	1.58
7	17	0.5	50	50	1.87
8	5	3.5	50	50	1.85
9	3	2	20	10	0.69
10	8	2	80	10	1.45
11	14	2	20	50	1.45
12	4	2	80	50	2.05
13	12	2	50	30	1.93
14	16	2	50	30	1.47
15	2	2	50	30	1.74
16	1	2	50	30	1.80
17	7	2	50	30	1.56
18	9	0.5	20	30	1.41
19	10	0.5	80	30	2.07
20	11	0.5	50	10	1.70
21	17	0.5	50	50	2.18

^1^ TPC—Total phenolic content; ^2^ GAE—gallic acid equivalents; dw—dry weight of medlar fruit.

**Table 2:** Estimated regression coefficients and analysis of variance of fitted second-order polynomial models for the examined parameters for physiologically ripe medlar fruit (PRMF) and consumable ripe medlar fruit (CRMF).

Source	Sum of Squares	df	Mean Square	F-Value	*p*-Value
^1^ TPC Optimization for PRMF					
Model	1.93	4	0.4836	40.69	<0.0001
A-Time	0.1233	1	0.1233	10.37	0.0074
B-Solid/solvent ratio	0.7069	1	0.7069	59.47	<0.0001
C-Ethanol concentration	0.7694	1	0.7694	64.73	<0.0001
C^2^	0.335	1	0.335	28.18	0.0002
Residual	0.1426	12	0.0119		
Lack of Fit	0.1068	8	0.0134	1.49	0.3692
Pure Error	0.0358	4	0.0089		
Cor Total	2.08	16			
^2^ R^2^	0.9313				
Adjusted R^2^	0.9084				
Predicted R^2^	0.8499				
^1^ TPC optimization for CRMF					
Model	1.73	3	0.5769	20.01	<0.0001
A-Time	0.0410	1	0.0410	3.45	0.088
B-Ethanol concentration	0.9275	1	0.9275	32.16	<0.0001
C-Solid–liquid ratio	0.6261	1	0.6261	21.71	0.0004
B^2^	0.1772	1	0.1772	6.15	0.0277
Residual	0.3749	13	0.0288		
Lack of Fit	0.2382	9	0.0265	0.7744	0.657
Pure Error	0.1367	4	0.0342		
Cor Total	2.11	16			
^2^ R^2^	0.822				
Adjusted R^2^	0.7809				
Predicted R^2^	0.7062				

^1^ TPC—Total phenolic content; ^2^ R^2^—coeficient of determination.

**Table 3:** Experimental and predicted values by response surface methodology (RSM) under conditions estimated as optimal total phenolic extraction and prediction capacity of the RSM model. ^a^ Mean values (*n* = 3) followed by different letters differ significantly, at *p* < 0.05.

TP ^1^	PRMF Extract	CRMF Extract
Experimental values (mg GAE/g dw) ^2^	1.95 ± 0.16 ^a^	2.52 ± 0.17 ^a^
Values predicted by RSM (mg GAE/g dw)	2.06 ± 0.11	2.10 ± 0.17
Confidence Interval 95%	1.92–2.21	1.90–2.29
Tolerance Interval 95%	1.52–2.61	1.29–2.91

^1^ Total phenolics; ^2^ GAE—gallic acid equivalents; dw—dry weight of medlar fruit.

**Table 4:** Total phenolic content (TPC) and individual phenolic compounds in the optimized lyophilized extracts.

Polyphenolic Compound	PRMF	CRMF
TPC (mg GAE/g dwle) ^1^	4.35 ± 0.04 ^a2^	4.06 ± 0.12 ^b^
	μg/g dwle	
Rutin	44.21 ± 1.17 ^b2^	54.54 ± 1.45 ^a^
Hyperoside	0.03 ± 0.00	0.03 ± 0.00
Quercetin	0.05 ± 0.00	0.04 ± 0.00
Chlorogenic acid	20.67 ± 0.78 ^b^	24.49 ± 0.84 ^a^
Caffeic acid	34.07 ± 1.11	32.50 ± 1.03
Isoquercitrin	139.96 ± 3.15 ^a^	114.67 ± 2.87 ^b^
Epicatechin	9.33 ± 0.17 ^b^	13.04 ± 0.21 ^a^
Procyanidin B2	38.46 ± 0.47 ^a^	23.56 ± 0.36 ^b^

^1^ GAE—Gallic acid equivalents; dwle—dry weight of lyophilized extract; ^2^ results are presented as mean ± standard deviation. Mean values (*n* = 3) followed by different letters differ significantly, at *p* < 0.05.

**Table 5:** Antioxidant activity of PRMF and CRMF optimized lyophilized extracts and standard.

Sample	DPPH (mg/mL)	ABTS^+^ (mg/mL)	FRAP (µmol Fe^2+^/g)
PRMF	3.25 ± 0.01 ^b1^	1.26 ± 0.03 ^b^	33.29 ± 0.13 ^b^
CRMF	3.27 ± 0.00 ^b^	1.33 ± 0.02 ^b^	31.51 ± 0.41 ^b^
Vitamin C	0.0045 ± 0.00 ^a^	0.0023 ± 0.00 ^a^	0.016 ± 0.00 ^a^

^1^ Results are presented as mean ± standard deviation. Mean values (*n* = 3) followed by different letters differ significantly at *p* < 0.05.

## Data Availability

The original contributions presented in this study are included in the article/Supplementary Material. Further inquiries can be directed to the corresponding authors.

## Supplementary Materials

The following supporting information can be downloaded at https://www.mdpi.com/article/10.3390/plants15081169/s1: Figure S1. Chromatogram of CRMF recorded at 350 nm: 1—chlorogenic acid, 2—caffeic acid, 3—rutin, 4—hyperoside, 5—isoquercitrin, 6—quercetin; Figure S2. Chromatogram of PRMF recorded at 350 nm: 1—chlorogenic acid, 2—caffeic acid, 3—rutin, 4—hyperoside, 5—isoquercitrin, 6—quercetin; Figure S3. Chromatogram of CRMF recorded at 260 nm: 6—epicatechin, 7—procyanidin B2; Figure S4. Chromatogram of PRMF recorded at 260 nm: 6—epicatechin, 7—procyanidin B2.
