# Supplementary material for: Underutilized Medlar (Mespilus germanica L.) Fruit: Polyphenol Extraction Optimization, Chemical Profiling, and In Vitro Pharmacological Evaluation
Source: Plants (Basel). 2026 Apr 10;15(8):1169. doi: 10.3390/plants15081169 (PMC13119120; doi:10.3390/plants15081169)
Supplement: Supplementary file 1 [file plants-15-01169-s001.zip › plants-4185954-Supplementary material.pdf]

SUPPLEMENTARY MATERIAL

Underutilized Medlar (*Mespilus germanica* L.) Fruit: Polyphenol Extraction Optimization, Chemical Profiling, and In Vitro Pharmacological Evaluation

Nenad Mićanović[1*], Nada Ćujić Nikolić[2], Jelena Živković[2], Katarina Šavikin[2], Nemanja Krgović[2], Jelena Popović-Đorđević[1*]

1 University of Belgrade, Faculty of Agriculture, Nemanjina 6, 11080 Belgrade, Serbia
2 Institute for Medicinal Plants Research "Dr. Josif Panˇci´c", Tadeuša Koš´cuška 1, 11000 Belgrade, Serbia; ncujic@mocbilja.rs (N.Ć.N.); jzivkovic@mocbilja.rs (J.Ž.); ksavikin@mocbilja.rs (K.Š.); nkrgovic@mocbilja.rs (N.K.)
* Correspondence: micanovic.nenad@gmail.com (N.M.); jelenadj@agrif.bg.ac.rs (J.P.-Đ.)

TABLE OF CONTENTS

**Figure S1**.	Chromatogram of CRMF recorded at 350 nm: 1 – chlorogenic acid, 2 – caffeic acid, 3 – rutin, 4 – hyperoside, 5 – isoquercitrin, 6 – quercetin
**Figure S2**	Chromatogram of PRMF recorded at 350 nm: 1 – chlorogenic acid, 2 – caffeic acid, 3 – rutin, 4 – hyperoside, 5 – isoquercitrin, 6 – quercetin
**Figure S3**	Chromatogram of CRMF recorded at 260 nm: 6 – epicatechin, 7 – procyanidin B2
**Figure S4**	Chromatogram of PRMF recorded at 260 nm: 6 – epicatechin, 7 – procyanidin B2

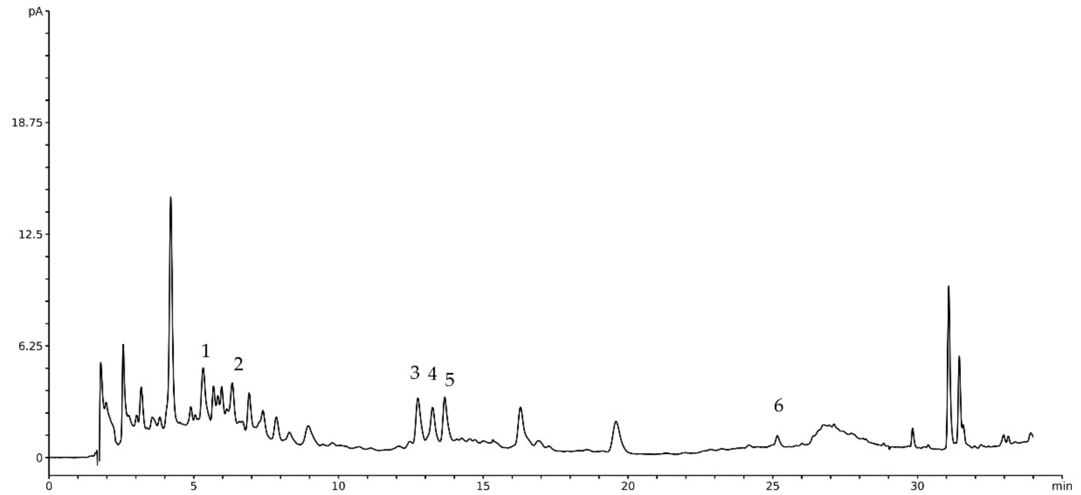

Figure S1. Chromatogram of CRMF recorded at 350 nm: 1 – chlorogenic acid, 2 – caffeic acid, 3 – rutin, 4 – hyperoside, 5 – isoquercitrin, 6 – quercetin

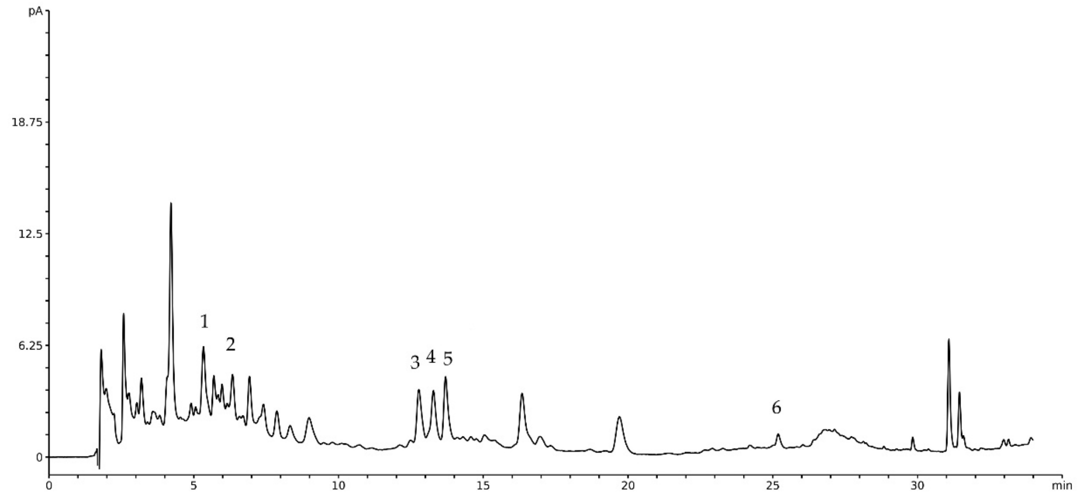

Figure S2. Chromatogram of PRMF recorded at 350 nm: 1 – chlorogenic acid, 2 – caffeic acid, 3 – rutin, 4 – hyperoside, 5 – isoquercitrin, 6 – quercetin

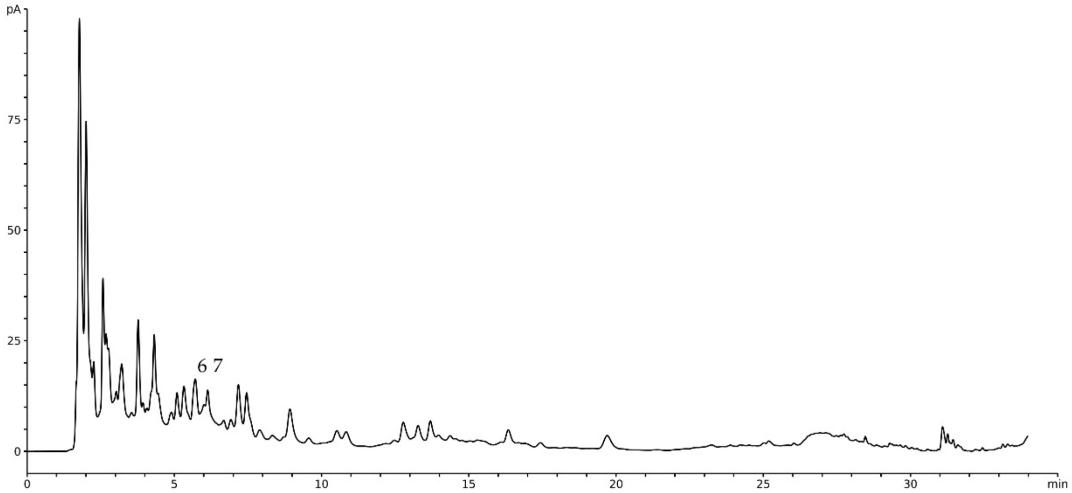

Figure S3. Chromatogram of CRMF recorded at 260 nm: 6 – epicatechin, 7 – procyanidin B2

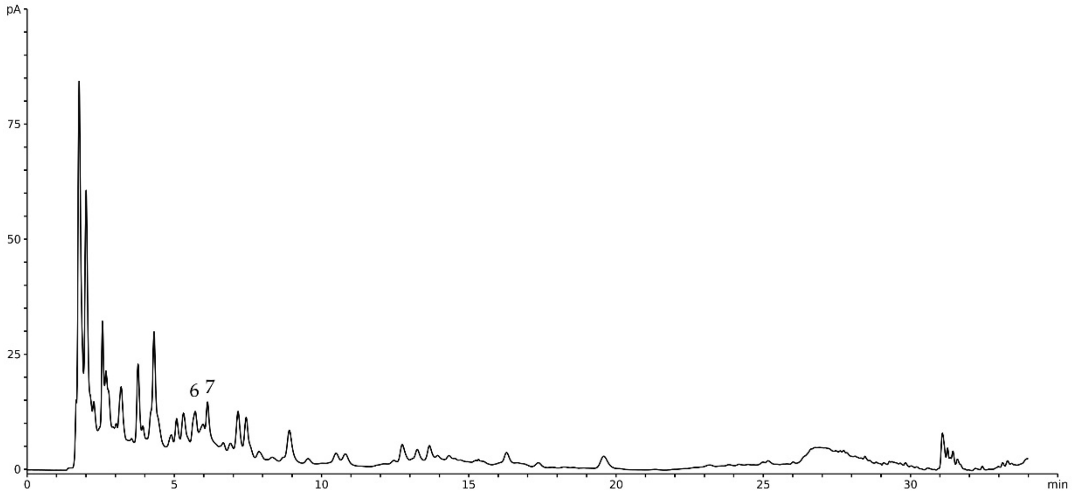

Figure S4. Chromatogram of PRMF recorded at 260 nm: 6 – epicatechin, 7 – procyanidin B2
